# Healthcare resource utilization and direct costs of transfusion-dependent thalassemia patients in Dubai, United Arab Emirates: a retrospective cost-of-illness study

**DOI:** 10.1186/s12913-022-07663-6

**Published:** 2022-03-05

**Authors:** Shaikha Alshamsi, Samer Hamidi, Hacer Ozgen Narci

**Affiliations:** 1 School of Health and Environment Studies, Hamdan Bin Mohammed Smart University, Dubai, UAE; 2 Department of Health Management, Istinye University, Istanbul, Turkey

**Keywords:** Healthcare resource utilization, Direct costs, Transfusion-dependent thalassemia, Health policy, Dubai, United Arab Emirates

## Abstract

**Background:**

Patients with transfusion-dependent thalassemia (TDT) require lifelong blood transfusions and iron chelation therapy. Thus, patients afflicted with TDT often have to undergo blood transfusion and iron chelation therapy, which causes a major economic burden on them. However, this topic has not been reported in Dubai, United Arab Emirates (UAE). Hence, this study aimed to evaluate healthcare resource utilization and associated direct costs related to patients with TDT in Dubai, UAE.

**Methods:**

For this study, a retrospective prevalence-based cost-of-illness analysis based on the UAE healthcare system and patient perspectives was conducted among patients with TDT treated at the Dubai Thalassemia Center in 2019. Information regarding healthcare resource utilization and direct medical costs was collected from the billing system connected to the electronic medical record system. Patients and their families were interviewed for direct non-medical cost estimations.

**Results:**

A total of 255 patients with TDT were included in the study. The mean annual direct medical cost was estimated at AED 131,156 (USD 35,713) (95% CI: 124,735 – 137,578). The main driver of the medical cost for the participants as iron chelation therapy AED 78,372 (95% CI: 72,671 – 84,074) (59.8%), followed by blood transfusions, which accounted for AED 34,223 (95% CI: 32,854 – 35,593) 26.1% of the total direct medical costs. The mean annual direct non-medical costs was AED 2,223 (USD 605) (95% CI: 1,946 – 2,500). Age (*p* < 0.001), severe serum ferritin levels (*p* = 0.016), the presence of complications (*p* < 0.001), and the type of iron chelation therapy (*p* < 0.001) were significant predictors of higher direct medical costs incurred by the participants.

**Conclusion:**

Transfusion-dependent thalassemia poses a substantial economic burden on the healthcare system, patients, and their families. Our results show that the highest medical cost proportion was due to iron chelation therapy. In this regard, efforts must be made to improve the patients’ acceptance and satisfaction with their iron chelation therapy to increase their compliance and improve the effectiveness of treatment, which could play an essential role in controlling the economic burden of this disease. Moreover, greater support is essential for families that suffer catastrophic out-of-pocket expenses.

**Supplementary Information:**

The online version contains supplementary material available at 10.1186/s12913-022-07663-6.

## Background

Hemoglobin disorders are the most widespread inherited diseases across the world; it was estimated that there were approximately 399 million thalassemia carriers in 2019 [1]. Hemoglobinopathies are among the most common monogenic disorders among UAE nationals [2, 3]. The most frequently observed hemoglobinopathy among UAE nationals is the α-thalassemia trait, with the highest carrier frequency worldwide (49%) [4]. The rate of prevalence of β-globin defects in the UAE is 8.5%, which constitutes a significant health problem, as the vast majority of β-thalassemia mutations in the UAE are very severe [5]. Hemoglobin disorders represent a prominent health concern in the UAE, especially in Dubai, where they significantly affect morbidity and mortality of the total population. The estimated cost of thalassemia treatment for a child with TDT up to age 16 is approximately (UAE Dirham) AED 1.2 million (USD 327,000) [6]. Therefore, such hemoglobin disorders create an economic burden on the healthcare system and a country’s financial resources, even with screenings, charity funding, and awareness campaigns. Despite the decreasing number of individuals with thalassemia major, the thalassemia carrier population has gradually increased as the population in UAE increased [7]. The high prevalence of thalassemia carriers in the country is still a significant health problem, as there is a 25% risk of having a baby with TDT if a marriage between carriers occurs. Carrier marriages substantially contribute to an increase in the thalassemia major population [8].

Long-term treatment for patients with TDT requires specialist and multidisciplinary input, which is expensive and tends to increase with patient’s age [9, 10]. According to a report by Research and Markets, global treatment cost for thalassemia was about US$ 842.0 million in 2017 and is expected to increase by 7.9% from 2018 to 2026 due to increasing prevalence of thalassemia and for the cause of raising the awareness of the disease worldwide [11]. The costs of TDT treatment varied across countries [12–16] and ranged from USD 563 to USD 128,062 [15, 16]. Different factors could cause this variation, including the age of patients, different treatment regimens, and patients' clinical characteristics.

Most of the cost-of-illness studies on TDT have revealed that the costs of lifelong conventional therapy for thalassemia are high and place an enormous economic burden on the healthcare system as well as the patients [16–18]. This massive economic burden is majorly attributed to blood transfusions and iron chelation therapy (ICT) [16, 19–23]. Other studies have also revealed that blood transfusion and nursing services and comprise the lion share of the total medical expenditure [24], and that ICT and in-hospital stay are the most expensive components of direct medical cost [12].

Furthermore, it has been observed that ICT cost estimates are higher in the first year than in the following years [25]. Increasing age is associated with increased healthcare costs and healthcare resource utilization (HCRU) [14, 26]. Major complications of hemochromatosis associated with TDT increase with age [27, 28] thereby increasing direct medical costs [29].

Ongoing TDT management poses a substantial economic burden on the healthcare system, and the cost incurred by the families of TDT patients could lead to catastrophic healthcare expenditures. According to the World Bank defines a financial catastrophe as out-of-pocket payments exceeding 10% of the total household income [30]. In India, in families with thalassemia patients, 38.8% of the family income is spent on the treatment of the disease annually [31], whereas in Pakistan, the total monthly healthcare cost was Rs. 9626 per patient. This cost puts a huge economic burden on families with TDT patients [17]. In Sri Lanka, a study concluded that thalassemia management could require about 5% of the country’s total health budget, and the mean healthcare household expenditure was USD 206 annually, and 26.5% of the families experienced catastrophic level of healthcare expenditure in the care of their children [32]. In Malaysia, families spend USD 678 per patient per year, which is approximately 3.3% of the monthly household income [33].

The HCRU and associated direct costs for patients with TDT in Dubai, UAE are not known as of yet. Thus, the objective of our study was to assess the economic burden on both the healthcare system and the TDT patients and their families in Dubai by estimating the HCRU and direct medical and non-medical costs and identify their determinants to aid decision-makers in managing and controlling the economic burden of the TDT effectively.

## Methods

### Data source

The primary data source for direct medical estimation was the billing system that connected with the patients’ medical records (SALAMA) of TDT patients treated at Thalassemia Center in Dubai from January 1, 2019, to December 31, 2019. Patient-level data on HCRU collected during a one-year period and unit cost per healthcare service for each TDT patient were extracted from the database. Information regarding sociodemographic characteristics [age, sex, nationality, and monthly income] was collected by interviewing patients and their families, whereas clinical characteristic information [type of TDT, type of ICT, ferritin level, presence of complications, and splenectomy] was extracted from the patients’ records. The HCRU and associated medical costs were categorized into laboratory investigations, radiology and other diagnostic investigations, consultations, blood transfusion, ICT, other medications, and surgical and other therapeutic procedures. Hospitalization services were not included in the study because more than 60% of TDT patients lived outside Dubai and might have sought medical care in their respective cities, which might have caused underestimation. Information regarding the direct non-medical cost (transportation cost) was collected through a self-administered questionnaire from December 4, 2019, to February 6, 2020. The average number of visits per month, mode of transport used, and journey distance were obtained for all patients. The transportation cost of the duration of a year was extrapolated by calculating the number of visits per month.

### Study design and sample

A prevalence-based cost-of-illness study design was used to characterize HCRU, estimate the direct medical and non-medical costs from the perspective of the healthcare system and the patients, and identify the factors affecting HCRU and direct medical costs. The HCRU and direct costs of TDT were estimated using a bottom-up, prevalence-based approach. In this regard, the HCRU and the direct medical and non-medical costs of TDT patients per year were estimated.

The Dubai Thalassemia Center has approximately 850 patients (42.5%), and approximately 450 (52.9%) were on regular blood transfusions [34]. All patients who had to undergo regular blood transfusions for at least one year, were included in the study.

### Sample size calculation

A total of 314 TDT patients were eligible target population registered at the Dubai Thalassemia Center during the study period (December 4, 2019, to February 6, 2020). The sample size was calculated based on the following formula [35]:

$$n=\frac{\left(\frac{P\left[1-P\right]}{\frac{{A}^{2}}{{Z}^{2}}+\frac{P\left[1-P\right] }{N}}\right)}{R}$$

where:

*n* = sample size required.

N = number of people in the population (314 patients).

P = estimated variance in the population, as a decimal (0.5 for 50–50 variability).

A = Precision desired, expressed as a decimal (0.03 for 3%).

Z = Based on confidence level: 1.96 for 95% confidence.

R = Estimated response rate, as a decimal (95% according to the pilot study).

According to the previous equation and based on a 3% margin of error with a 95% level of confidence for continuous data [36], a total of 243 TDT patients were required to perform the study. To reduce the sampling error due to non-response respondents, a 10% of the sample size was increased, and a total of 267 patients were interviewed using a random sampling method.

### Data collection

The primary data were retrieved from the billing system connected to the electronic medical records system of the facility. Associated medical costs and HCRU were extracted for a one-year period from January 2019 to December 2019. Surveys were distributed to 267 patients or their parents during blood transfusion sessions to collect secondary data related to direct non-medical costs (see Additional file 1).

### Cost and healthcare resource utilization calculation

Information on HCRU for each patient and the unit price for each service was obtained from the electronic medical records system. Cost summations for all resources were calculated per patient per year according to the type of services used per patient per year (laboratory investigations, radiology investigations, consultations, ICT, blood transfusions, other medications, and surgical and other therapeutic procedures).

The total direct medical cost was calculated by multiplying the total number of HCRUs by the unit cost for each healthcare resource. The summation of costs from all resources utilized within one year for each patient was considered to attain the total medical costs for a patient. The average annual direct medical cost per patient was calculated by dividing the total annual costs by the total number of patients (255 patients) included in the study.

For direct non-medical costs, the average number of visits, mode of transport used, and distance covered by the patients to reach the hospital and returning, were obtained for all patients. For public transport, the total fare was used to calculate the transportation cost per visit. For travel by private car, the transportation cost per visit was calculated from the journey covered in miles multiplied by the average cost per mile, allowing for fixed costs, depreciation, and running costs. No data were available for the UAE or neighboring countries on the average cost of driving per mile, so the data were adopted from American Automobile Association data [37]; an average figure of 61.88 cents/mile was used, which is equal to 2.27 AED/mile (based on 15,000 miles/year). Furthermore, the out-of-pocket cost burden was calculated to assess the effects of any financial catastrophe associated with TDT on patients. Seventeen patients reported zero income and were excluded from the calculations, as their income denominators were zero.

To calculate the ratio of out-of-pocket household payments for healthcare (transportation costs) to household income, the following formula was used:

Annual household out-of-pocket expenditure for healthcare (transportation cost)/ total annual household income * 100.

### Data analysis

Descriptive analysis was performed using the mean and standard deviation for continuous variables with normally distributed data and using the median and interquartile range for skewed data, while categorical variables were reported as absolute and relative frequencies. The normality of distribution was assessed using the One-Sample Kolmogorov – Smirnov test for all variables. To determine the significance of the difference, the Mann–Whitney U test or Kruskal–Wallis H test was used for continuous variables with non-normally distributed data, and the chi-square test for categorical variables with non-normally distributed data.

To study the effect size of a chi-square independence test, Cramer’s V test (for nominal variables) was used. Spearman correlation was used to study the association between continuous non-normally distributed variables, and Goodman and Kruskal's lambda was used to determine the association between two nominal variables (see Additional file 2).

Regression analysis was implemented to determine the factors affecting HCRU and the direct medical costs associated with TDT. A negative binomial regression model was used as the appropriate model for discrete data with overdispersion to identify predictors associated with HCRU. A generalized linear model (GLM) with a log-link function and gamma distribution was selected to study the significant factors affecting the direct medical costs of TDT. Methods of selecting the independent variables and evaluating the assumptions of regression, such as collinearity, independence of residuals, linearity, outliers, and homoscedasticity, are mentioned in detail in the Additional file 2. All inferential tests were two-sided, and a 95% significance level was used. Statistical significance was set at *p* < 0.05. All analyses were conducted using Stata/IC 16 (StataCorp, College Station, Texas 77,845 USA). According to the guidelines outlined by International Society of Pharmacoeconomics and Outcomes Research (ISPOR), the sample mean cost is considered the most appropriate measure for healthcare policymakers. The average cost was reported despite the non-normal distribution of the cost data [38].

## Results

### Sociodemographic and clinical characteristics of TDT patients

A total of 267 patients were included in the study; 255 agreed to participate in this study, with a response rate of 96.2%. As indicated in Table 1, the highest proportion of patients (73.3%) were in the > 18 years age group. More than half of the patients (65.5%) were non-UAE nationals, and 25.1% of patients had the lowest annual household income quartile. The major type of thalassemia present in the sample was β-thalassemia major (90.6%), and 82.2% of patients had disease complications. More than half of the patients (67.8%) had bone complications, whereas cardiac complications were the least common type of complications associated with TDT (7.8%). Four different ICTs were used in TDT patients: Deferasirox (DFX), deferiprone (DFP), deferoxamine (DFO), and combined therapy. Among the patients with TDT, DFX was the most common ICT used (71.0%), followed by DFO (13.7%), and the least used was DFP (4.3%). More than half of the patients (62.7%) had severe serum ferritin levels (≥ 2000 ng/ml).

**Table 1:** Sociodemographic and clinical characteristics of TDT patients

Characteristics	Adult	Children	Total
Age in years, mean(SD)			25.0 (9.8)
Age, n(%)			
≤ 18	-	68(26.7%)	
> 18	187(73.3%)	-	
Sex, n(%)			
Male	97(76.4%)	30(23.6%)	127(49.8%)
Female	90 (70.3%)	38(29.7%)	128(50.2%)
Household annual income (AED), n(%)			
1st quartile (≤ 37,200)	55(29.4%)	9(13.2%)	64(25.1%)
2nd quartile (> 37,200– 96,000)	50 (26.7%)	21(30.9%)	71(27.9%)
3rd quartile (> 96,000 – 192,000)	42 (22.5%)	18(26.5%)	60(23.5%)
4th quartile (> 192,000)	40 (21.4%)	20(29.4%)	60(23.5%)
Nationality, n(%)			
UAE	59(31.6%)	29(42.6%)	88(34.5%)
Non-UAE	128 (6.4%)	39(57.4%)	167(65.5%)
Disease type, n(%)			
β-thalassemia major	172(92.0%)	59(86.7%)	231(90.6%)
β-thalassemia intermedia	7(3.7%)	7(10.3%)	14(5.5%)
Hemoglobin E/β-thalassemia	7(3.7%)	1(1.5%)	8(3.1%)
α-thalassemia intermedia	1(0.5%)	1(1.5%)	2(0.8%)
ICT, n(%)			
DFX	125(66.8%)	56(82.4%)	181(71.0%)
DFP	10(5.3%)	1(1.5%)	11(4.3%)
DFO	26(13.9%)	9(13.2%)	35(13.7%)
Combined therapy	26(13.9%)	2(2.9%)	28(11.0%)
Ferritin level, n(%)			
< 2000 ng/ml	70(37.4%)	25(36.8%)	95(27.3%)
≥ 2000 ng/ml	117(62.6%)	43(63.2%)	160(62.7%)
History of splenectomy, n(%)			
No	162(86.6%)	67(98.5%)	229(89.8%)
Yes	25(13.4%)	1(1.5%)	26(10.2%)
Number of complications, n(%)			
No complication	5(2.7%)	39(57.4%)	44(17.2%)
One complication	83(44.4%)	25(36.8%)	108(42.4%)
Two complications	69(36.9%)	4(5.9%)	73(28.6%)
Three or more complications	30(16.0%)	0 (0%)	30(11.8%)
Cardiac complications, n(%)			
No	169(90.4%)	66(97.1%)	235(92.2%)
Yes	18(9.6%)	2(2.9%)	20(7.8%)
Hepatic complications, n(%)			
No	146(78.1%)	67(98.5%)	213(83.5%)
Yes	41(21.9%)	1(1.5%)	42(16.5%)
Endocrine system complications, n(%)			
No	85(45.5%)	57(86.8%)	142(55.7%)
Yes	102(54.5%)	11(13.2%)	113(44.3%)
Bone complications, n(%)			
No	35(18.7%)	47(69.1%)	82(32.2%)
Yes	152(81.3%)	21(30.9%)	173(67.8%)

*ICT* iron chelation therapy, *DFX* Deferasirox, *DFP* deferiprone, *DFO* deferoxamine

### Healthcare resource utilization associated with TDT

The highest annual median number of HCRU reported for TDT patients was 1,104 for other medications, followed by ICT (1,023); whereas the lowest was in the surgical and other therapeutic procedures with the (0) procedure.

The highest annual median of ICT per patient was 2,296 tablets for DFP therapy (orally administered three times per day), followed by a combined therapy (2,143 tablets and vials) (mix of two or three ICT- intravenous and orally administered), DFX therapy (1,020 tablets) (oral administration; one tablet per day), and the least used was DFO therapy (398 vails) (intravenous and administered over 8–24 h; 5–7 days per week) (Table 2).

**Table 2:** Distribution of the annual HCRU in TDT treatment per patient per year (2019)

Healthcare resource utilization	Median	Average/ patient	Min	Max
**Laboratory investigations**	113	120	55	376
**Radiology and other diagnostic investigations**	3	4.0	0	23
**Blood transfusion**	41	41	7	82
**Physician consultation**	6	10	0	93
**Surgical and other therapeutic procedures**	0	1	0	35
**Other medications**	1104	717	20	5,632
**ICT (total)**	1,023	1,220	5,846	311,096
DFX	1,020	1,032	91	3,523
DFP	2,296	2,599	476	5,832
DFO	398	782	0	3,822
Combined therapy	2,143	2,443	373	5,846

*ICT* iron chelation therapy, *DFX* deferasirox, *DFP* deferiprone, *DFO* deferoxamine

Adult patients and patients with non-UAE nationality had significantly higher annual median number of radiology investigations than children and those with UAE nationality (*p* = 0.024 and *p* < 0.001, respectively). Moreover, male patients aged > 18 years had a significantly higher annual median number of blood transfusion units than female and child patients (*p* < 0.001 and *p* < 0.001, respectively). Healthcare resource utilization differed significantly across patients with TDT with different clinical characteristics. Patients with α-thalassemia intermedia and those with severe serum ferritin levels (> 2000 ng/ml) underwent a significantly higher median number of laboratory investigations than those with other thalassemia types and with mild-to-moderate ferritin levels (*p* = 0.035 and *p* = 0.040, respectively). Patients treated with DFO had the lowest annual median number of blood transfusions (*p* = 0.025). Patients treated with DFO also had a significantly higher median number of consultation services than those treated with other iron chelators (*p* < 0.001). Patients diagnosed with disease complications had a significantly higher median number of radiology investigations and blood transfusion units than those without it (*p* < 0.001 and *p* < 0.001, respectively). Splenectomized patients had significantly higher annual median numbers of radiology investigations (*p* = 0.025) and consultations (*p* = 0.021) and a substantially lower annual median number of blood transfusions than non-splenectomized patients (*p* = 0.048) (see Additional file 3).

Analysis with the chi-square test identified a significant and strong association between the type of iron chelator used and patient age (χ^2^ (3) = 9.19, *p* = 0.023, Cramer’s V = 0.187). Deferasirox therapy was the most common iron chelator used across the two age groups, and its highest proportion of usage was in the age group of > 18 years. There was a significant and very strong association between the type of iron chelator used and ferritin levels in patients with TDT (χ^2^ (3) = 23.47, *p* < 0.001, Cramer's V = 0.292). Treatment with DFP was significantly associated with mild-to-moderate serum ferritin levels (< 2000 ng/ml). Contrastingly, treatment with other iron chelators was significantly associated with severe serum ferritin levels (≥ 2000 ng/ml). No significant associations were found between iron chelators and other characteristics of TDT patients, such as sex, disease types, presence of complications, and history of splenectomy (see Additional file 3).

### Predictors of healthcare resource utilization associated with TDT

Negative binomial regression identified one statistically significant positive predictor and one significant negative predictor for the utilization of laboratory investigations. A severe serum ferritin level (≥ 2000 ng/ml) was associated with an expected increase in the rate of laboratory investigation utilization by a factor of 1.13, whereas hemoglobin E/β-thalassemia was associated with an expected decrease in the laboratory investigation utilization rate by a factor of 0.71.

For the utilization of radiology investigations, negative binomial regression identified one statistically significant positive predictor of patient age. With every one-year increase in TDT patient age, there was an increase in the utilization of radiology and other diagnostic investigations by a factor of 1.014.

Two statistically significant positive predictors and three statistically significant negative predictors related to the number of blood transfusion units were also identified. With every one-year increase in TDT patient age, an increase in the number of blood transfusion units by a factor of 1.017 was noted. Moreover, compared with no complications, the presence of complications was associated with an expected increase in the number of blood transfusion units by a factor of 1.43; contrastingly, female sex was associated with an expected decrease in the number of blood transfusion units by a factor of 0.84 compared to male sex. Splenectomy was associated with an expected decrease in the number of blood transfusion units by a factor of 0.77. Additionally, hemoglobin E/β-thalassemia was associated with an expected decrease in the number of blood transfusion units by a factor of 0.86 compared to β-thalassemia major.

For the utilization of consultation services, negative binomial regression identified three statistically significant positive predictors. The use of DFO and combined therapy was associated with expected increases in the number of consultation services utilized by factors of 2.73 and 1.77, respectively, compared to DFX. Furthermore, splenectomy was associated with an expected increase in the number of consultation services by a factor of 1.47 when compared to no splenectomy (Table 3).

**Table 3:** Incident rate ratio for healthcare resource utilization in TDT

Healthcare services	IRR*	Robust Std. error	*P* >|z|*	95% CI
**Laboratory investigations**				
Disease types				
Hemoglobin E/β-thalassemia (ref. β-thalassemia major)	0.71	0.06	< 0.001	0.60 – 0.82
Ferritin level				
≥ 2000 ng/ml (ref. < 2000 ng/ml)	1.13	0.04	< 0.001	1.06 – 1.21
** Radiology investigations**				
Age	1.014	0.006	0.023	1.001 – 1.026
** Blood transfusions**				
Age	1.017	0.003	< 0.001	1.012 – 1.021
Sex				
Female (ref. male)	0.84	0.03	< 0.001	0.79 – 0.90
Disease types				
Hemoglobin E/β-thalassemia (ref. β-thalassemia major)	0.86	0.06	0.028	0.75 – 0.98
Complications				
Yes	1.43	0.12	< 0.001	1.22 – 1.69
Splenectomy				
Yes	0.77	0.04	< 0.001	0.69 – 0.87
** Consultations**				
ICT				
DFO	2.73	0.50	< 0.001	1.90 – 3.90
Combined therapy (ref. deferasirox)	1.77	0.36	0.005	1.19 – 2.62
Splenectomy				
Yes	1.47	0.27	0.035	1.03 – 2.09

*IRR* incidence rate ratio, *ICT* iron chelation therapy, *DFX* Deferasirox, *DFP* deferiprone, *DFO* deferoxamine

### Direct medical costs associated with TDT

Direct medical expenditures include laboratory investigations, radiology investigations, ICT, other medications, blood transfusions, consultations, and surgical and other therapeutic procedures. The distribution of the direct medical costs associated with TDT is presented in Table 4. The mean total annual direct medical cost per patient was AED 131,156 (95% CI: 124,735 – 137,578), of which ICT was the most expensive component (AED 78,372; 95% CI: 72,671 – 84,074), followed by blood transfusions (AED 34,223; 95% CI: 32,854 – 35,593), laboratory investigations (AED 9,069; 95% CI: 8,695 – 9,443), other medications (AED 4,414; 95% CI: 3,244 – 5,584), consultations (AED 2,142; 95% CI: 1,800 – 2,483), radiology and other diagnostic investigations (AED 1,754; 95% CI: 1,527 – 1,982), and finally, surgical and other therapeutic procedures (AED 1,182; 95% CI: 681 – 1,683). According to the ICT used, the highest average cost of ICT per patient was paid for combined therapy (AED 91,586), followed by DFX (AED 88,929) and DFP (AED 31,123), whereas the least was paid for DFO (AED 28,058).

**Table 4:** Direct medical costs associated with TDT treatment by the type of service per patient per year (*N* = 255)

Service type	Average cost	Median	Min	Max	Total cost / year
**Laboratory investigations**	9,069	8,182	4,849	28,882	2,312,595
**Radiology investigations**	1,754	1,125	0	10,533	447,326
**ICT**	78,372	75,842	704	278,334	19,984,956
DFX	88,929	85,861	2,505	278,334	16,096,176
DFP	31,123	20,855	2,827	104,569	432,348
DFO	28,058	22,825	0	122,582	982,027
Combined therapy	91,586	83,630	18,508	147,234	2,564,405
**Other medications**	4,414	1,496	165	92,834	1,125,540
**Blood transfusions**	34,223	34,850	5,950	69,700	8,726,950
**Consultations**	2,142	1,246	0	21,265	546,115
**Surgical procedures**	1,182	0	0	33,004	301,361
**Total cost/year (AED) = 33,444,843**					
**Average cost/ patient/ year (AED) = 131,156**					
**Median cost/ patient/ year (AED) = 128,258**					
**Minimum cost/ patient/ year (AED) = 26,128**					
**Maximum cost/ patient/ year (AED) = 361,672**					

*ICT* iron chelation therapy, *DFX* deferasirox, *DFP* deferiprone, *DFO* deferoxamine

**Fig. 1:**
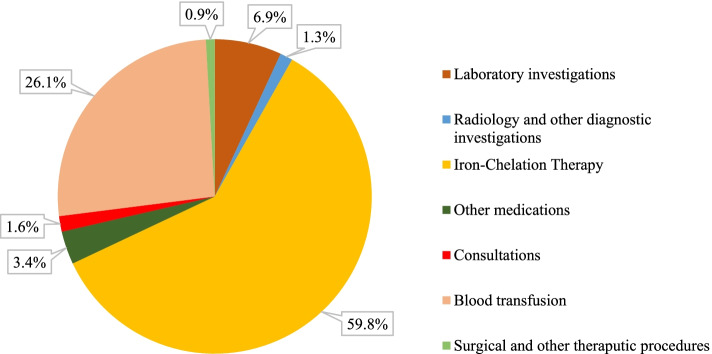
Distribution of the direct medical costs per TDT patient by medical service types, 2019

Concerning the distribution of direct medical costs per TDT patient according to the type of healthcare service (Fig. 1), ICT was the main cost driver (AED 78,372), accounting for more than half of the total annual direct medical costs (59.8%), followed by blood transfusions, which accounted for 26.1% (AED 34,223) of the total direct medical costs. The overall proportion of costs related to investigations was 8.2%, and that of other medications was 3.4%. The lowest proportion of costs was related to surgical and therapeutic procedures (0.9%).

In comparing patients of different ages, statistically significant differences in median total direct medical cost (*p* < 0.001) across the two age groups were found. The median total direct medical costs were found to be higher in adult patients (< 18 years old; AED 134,933) compared to child patients (*p* < 0.001). Moreover, male patients had a significantly higher median value of total medical costs (AED 136,426) than female patients (*p* = 0.004).

Concerning the distribution of total medical costs according to the ICT used, patients treated with combined therapy had a significantly higher median value of total medical costs (AED 153,059) than those treated with other ICTs (*p* < 0.001). Patients with complications had a significantly higher median value of medical costs (AED 134,859) than those without complications (*p* < 0.001). Moreover, patients who had to undergo splenectomy had a significantly lower median value of medical costs (AED 127,254) than patients who did not (*p* = 0.045). Direct medical costs were significantly weakly positively correlated with the number of complications (*p* = 0.202, *p* = 0.001), and patients’ age (*p* = 0.269, *p* < 0.001) (see Additional file 3).

### Predictors of direct medical costs for TDT.

According to the regression equation, with every one-year increase in the age of the TDT patient, there was a rise in the total direct medical costs of 1.4%. Female patients had 18.0% lower direct medical costs than male patients. Using DFX was associated with an expected increase in the direct medical costs of 50.0% compared to using DFO, and using a combined therapy was associated with an expected increase in the direct medical costs of 47.0% compared to using DFO. Hemoglobin E/β-thalassemia was associated with an expected decrease in direct medical costs of 34% compared to that of β-thalassemia major. Furthermore, having a severe serum ferritin level (≥ 2000 ng/ml) was associated with an expected increase in direct medical costs of 11.0%. Additionally, TDT-related complications were associated with an expected increase in direct medical costs of 30.0% (Table 5).

**Table 5:** GLM using gamma distribution with a log-link model of the total direct medical costs

Parameter	Coef	Robust Std. Err	P >|z|	95% CI
**Age**	0.014	0.00	< 0.001	0.008 – 0.020
**Sex**				
Female	-0.18	0.04	< 0.001	-0.26 – (-0.10)
**ICT**				
DFX	0.50	0.07	< 0.001	0.37 – 0.63
Combined therapy (ref. DFO)	0.47	0.09	< 0.001	0.29 – 0.64
**Disease type**				
Hemoglobin E/β-thalassemia (ref. β-thalassemia major)	-0.34	0.10	< 0.001	-0.53 – (-0.15)
**Ferritin level**				
≥ 2000 ng/ml	0.11	0.05	0.016	0.02 – 0.20
**Presence of complications**				
Yes	0.30	0.08	< 0.001	0.14 – 0.46

*GLM* generalized linear model, *ICT* iron chelation therapy, *DFX* deferasirox, *DFO* deferoxamine

### Direct non-medical costs associated with TDT

Blood transfusions should generally be administered at an interval of three–four weeks. In the Dubai Thalassemia Center, most of the patients with TDT (94.5%) had one visit for a blood transfusion every four weeks. In comparison, 5.5% of TDT patients visited a blood transfusion every three weeks. Moreover, most patients (92.5%) traveled to the Dubai Thalassemia Center by private car, while 7.1% used taxis and 0.4% used buses (see Additional file 3).

The mean distance traveled for private cars (miles, two-way) was 83.78 ± 83.97 miles. Using the American Automobile Association's estimate of gross motoring costs per mile, the annual mean cost of travel was AED 2,223 (95% CI: 1,946 – 2,500). According to patient age, the annual mean travel cost incurred by patients' parents was AED 2,137 (95% CI: 1,620 – 2,653), while the annual mean travel cost incurred by adult patients was AED 2,254 (95% CI: 1,923 – 2,584).

Bivariate analysis was conducted to identify the differences between the main outcome variable (transportation costs) and demographic characteristics of patients with TDT. The analysis showed that the number of visits per month and residence location of TDT patients were significantly associated with the annual transportation costs.

Patients who had one visit per month to Dubai Thalassemia Center had significantly lower median values of annual transportation costs than patients who had two visits per month (AED 1,015 and AED 2,410, respectively, *p* = 0.026). Patients coming from Oman had a significantly higher median annual transportation cost than other patients (AED 6,428, *p* < 0.001), followed by Fujairah and Abu Dhabi with approximately similar annual transportation costs (AED 4,026 and AED 4,229, respectively, *p* < 0.001). Contrastingly, the lowest median annual transportation costs were associated with patients who lived in Dubai (AED 600). No statistically significant differences were found in the annual transportation costs among the different transportation modes and annual income quartiles (see Additional file 3).

### Household cost burden

**Fig. 2:**
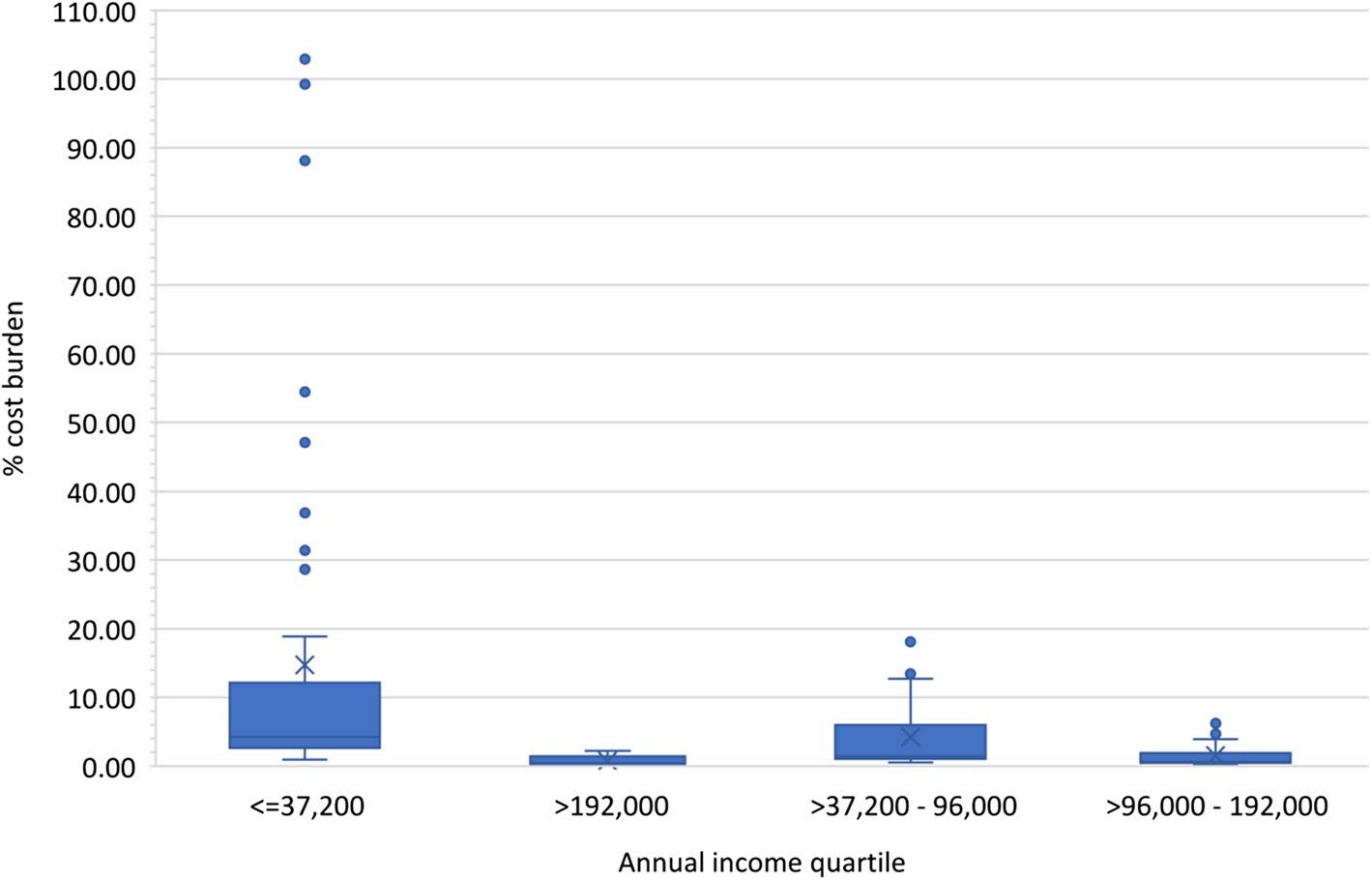
Relationship between annual household income quartile and cost burden

The median (IQR) household annual income was 96,000 AEDs (154,800). Patients were divided into four quartiles (Q1–Q4) based on their household income. Seventeen families reported no income and were excluded from the calculations. The median household cost burden was 1.3%, ranging from 0.08% to 102.9%. Twenty-eight patients (11.8%) experienced a catastrophic financial burden. Sixteen patients (34.0%) from the lowest income quartile and 12 patients (16.9%) in the second income quartile group experienced a cost burden of over 10%, which is considered a catastrophic cost burden despite the free medical available in Dubai Thalassemia Center (Fig. 2). Among all patients with catastrophic cost burden, 20 patients (71.4%) were from Oman, and only eight (28.6%) were from the UAE.

## Discussion

Most previous studies have revealed that the direct medical costs associated with TDT are high, and the management of the disease consumes considerable healthcare resources [26, 39]. The findings from this study provide estimates of HCRU and direct medical and direct non-medical costs associated with TDT, which, to the best of our knowledge, are the first estimates for Dubai. Patients having TDT usually have an iron overload that impairs the immune system and increases the risk of infection, illness, and organ damage such as cirrhosis, diabetes, heart disease, and hypogonadism [40]; therefore, TDT needs to be diagnosed and treated with appropriate utilization of healthcare resources.

Healthcare resource utilization in TDT management was described by different types of medical services used by TDT patients in 2019, such as laboratory services, radiology services, blood transfusion services, ICTs, and consultation services. According to ICT utilization evaluations, DFX was the most common ICT used among TDT patients (71%), whereas DFP had the highest average use, as DFP was taken three times a day, whereas DFX was taken once daily. Deferasirox is the first-line therapy for patients with TDT in the USA [41] and Europe [42].

The study revealed an association between patient age and serum ferritin levels with ICT. Deferasirox was the most common iron chelator used in the two groups of patients (≤ 18 and > 18 years). This finding could be explained by the fact that DFX is an effective long-term treatment for transfusion iron overload in pediatric and adult thalassemia patients [43]. However, the study showed that deferiprone was associated with mild-to-moderate serum ferritin levels (< 2000 ng/ml), whereas other ICTs were associated with severe serum ferritin levels (≥ 2000 ng/ml). This result indicates that DFP is a highly efficacious and safe chelation therapy for patients with TDT who are not compatible with other ICTs [44, 45].

For laboratory investigations, the bivariate analysis found that patients with severe serum ferritin levels (≥ 2000 ng/ml) had a higher number of laboratory investigations than patients with mild-to-moderate serum ferritin levels (< 2000 ng/ml). This finding is found by abiding by the guideline for the standard monitoring of patients with thalassemia, which mentioned that serum ferritin levels are used to monitor iron overload and should be measured at least every three months, and more frequent assessments may be considered if ferritin is not well controlled [46]. Additionally, patients diagnosed with hemoglobin E/β-thalassemia had significantly fewer laboratory investigations than others, which was also reported by a study conducted in Thailand [15]. The regression analysis revealed that an increase in the number of laboratory investigations was positively associated with severe serum ferritin levels (≥ 2000 ng/ml) and negatively associated with hemoglobin E/β-thalassemia. According to the guidelines for the standard monitoring of patients with thalassemia [46], patients with uncontrolled ferritin levels may need more frequent assessments, which leads to increased utilization of laboratory investigation services. Moreover, hemoglobin E/β-thalassemia was associated with an expected decrease in the number of laboratory investigations compared to β-thalassemia major, which has also been reported in Thailand [15]. Hemoglobin E/β-thalassemia is a thalassemia syndrome with a varied clinical spectrum [47]. This finding could be explained by the fact that the patients with hemoglobin E/β-thalassemia in this study had a less severe clinical presentation than patients with β-thalassemia major, thus justifying less use of healthcare services.

For radiology investigations, the study revealed that patients had complications resulting from regular blood transfusions and as consequences of iron overload; therefore, more radiological investigations are recommended to diagnose the endocrine, liver, bone, and cardiac complications associated with TDT. More radiology investigation utilization was found in patients who had to undergo splenectomies and patients with complications. Thrombocytosis and the risk of thromboembolic events are associated with splenectomy, and patients with complications require more radiological investigations to measure iron deposits in tissue, confirm the diagnosis, and monitor therapy [48].

The study also showed that expatriate patients had a significantly higher number of radiological investigations than patients residing in UAE. On comparing the number of complications in UAE patients and expatriate patients it was found that expatriate patients had a higher percentage of the number of complications than the UAE patients. Moreover, most of the patients who underwent splenectomy in this study were non-UAE national patients; therefore, expatriate patients used radiology services more often than UAE patients.

Regression analysis showed that patient age was a positive predictor of the utilization of radiological investigations. With the increasing age of TDT patients, more life-threatening complications are diagnosed, including severe thromboembolic events, pulmonary hypertension, and hepatocellular carcinoma [49]. Therefore, imaging services are increasingly being used to recognize and treat iron overload complications.

Most patients (94.5%) had one visit for blood transfusion every four weeks. On an average, blood transfusions were performed 15 times per year [13–17], which is the same as that reported in the USA and the UK [19, 50] and is considerably higher than that reported in Thailand (7.4 times per year) [15]. This variation was caused by the different patient age groups in the two studies. The number of blood transfusion units was higher in men than in women [51]. This finding could be explained by the fact that the normal hemoglobin level of males is higher than that of females, which suggests that males require a higher blood volume to achieve their normal hemoglobin level. Moreover, patients with cardiac complications received more blood units than those without cardiac complications [9, 52].

This result is in line with the Thalassemia International Federation (TIF) guidelines, which state that pre-transfusion hemoglobin should be between 9 and 10.5 g/dL. However, a higher target (11–12 g/dL) should be maintained for patients with cardiac complications or lower back pain close to the time of blood transfusion [9]. Patients who underwent splenectomy received fewer blood units than patients who did not. This finding is consistent with that of other studies [53, 54]. This result could be explained by the rationale for splenectomy in patients with TDT to decrease the volume of blood transfusions required to minimize iron overload [55, 56].

Patients treated with DFO had a significantly lower number of blood transfusion units than those treated with other ICTs. Contrastingly, another study showed that DFX reduced blood transfusion volume in relation to DFO [23]. This difference could be explained by the difference of sex in patients treated with DFO. More than half of our deferoxamine-treated patients were female, and as we mentioned earlier, female patients required less blood volume than male patients. Moreover, regression analysis revealed that age was a positive predictor of increased utilization of blood transfusion units. Similar to this study, another study found a linear relationship between age and blood transfusion units [57]. With increasing age, the number of blood transfusion units increased. A higher pre-transfusion hemoglobin level of 12 to 12 g/dl is appropriate for patients with cardiac complications or other medical conditions [9]; therefore, the presence of complications associated with TDT is a positive predictor of increased blood transfusion utilization. Similar to other studies [51, 54, 58], splenectomy and female sex were associated with an expected decrease in the number of blood transfusion units compared to male sex and no splenectomy. As mentioned earlier, the normal hemoglobin level for women is less than that of men; therefore, female patients required less blood volume to achieve normal hemoglobin levels than male patients. Moreover, the rationale for splenectomy was to reduce the volume of blood transfusion required to reduce iron overload. Hemoglobin E/β-thalassemia is a thalassemia syndrome of intermediate severity with a varied clinical spectrum, and there is some evidence that such patients can tolerate a low hemoglobin level [9], which is consistent with our results that showed that hemoglobin E/β-thalassemia was a predictor of a decrease in the number of blood transfusion units. Moreover, more than half of our hemoglobin E/β-thalassemia patients were female, which may have reduced the blood volume required.

Concerning the physician consultation rate, the univariate analyses revealed that splenectomized patients and patients treated with DFX had more physician consultations than non-splenectomized patients and those treated with other ICTs. These differences were caused by the fact that splenectomized patients tend to be more prone to clinical complications [59]. Patients treated with DFO, which has a very short half-life that requires repetitive injections and non-targeted distribution in tissues, can develop brain, lung, and kidney damage that can cause other problems, such as peripheral neuropathy and glandular and growth disorders [60]; this requires more frequent physician consultations than other ICTs. The regression analysis related to consultations revealed that DFO, combined treatment, and a history of splenectomy were associated with an expected increase in the number of consultation services. Despite the advantages of DFO, numerous deferoxamine-related problems have been reported, such as cardiovascular, respiratory, and visual field defects and deferoxamine-induced bone dysplasia. Therefore, patients treated with deferoxamine or combined treatments including deferoxamine could face more complications, which tend to increase the number of consultations. Moreover, splenectomized patients had a higher risk of infections such as sepsis and thrombotic complications [61] and therefore require more follow-ups and consultations than non-splenectomized patients.

Estimates of the direct medical costs associated with TDTs were almost consistent in different studies. Direct medical costs for patients with TDT are generally high [39], and most studies conclude that blood transfusions and ICT are the two main drivers of direct medical costs associated with TDT [13, 14, 23]. The study findings revealed that the total direct medical costs per TDT patient per year were AED 131,156 (USD 35,710) (USD 1 = AED 3.67), of which ICT was the most expensive cost component (59.8%), followed by blood transfusions (26.1%). The findings of this study have shown that the annual mean direct medical cost is in agreement with that found in other studies conducted in Western countries, such as Greece (EUR 32,054) [23] and Italy (EUR 31,883) [13], but lower than that found in studies conducted in the United States (USD 43,969 and USD 128,969) [16, 29]. Contrarily, our estimates are higher than those reported in middle- and low-income countries such as Turkey (USD 14,360) [12], Thailand (USD 563) [15], India (USD 1135) [31], and Iran (EUR 1,731) [24] (USD 7,287) [14]. The variations in the direct medical costs reported by previous studies because of the different inclusion criteria in each study, such as patients' ages and different types of thalassemia diseases and their severity, considerably affect the direct medical costs. Additionally, different years of study resulted in unreliable comparisons between the results. Furthermore, numerous monetary issues should be considered, such as the different purchasing power of currencies, inflation rate, and fluctuating exchange rates, especially in the imperfect healthcare market. Despite the variations in the direct medical costs associated with TDT, all studies concluded that TDT was associated with expensive direct medical costs.

Among the direct medical costs, the largest portion (59.8%, AED 78,372, USD 21,355) was paid for ICT, followed by blood transfusions, with 26.1% (AED 34,223, USD 9,325), and 8.2% (AED 10,823, USD 2,949) for laboratory and radiological investigations, 3.4% (AED 4,414) for other medications, 1.6% (AED 2,142) for consultations, and the least 0.9% (AED 1,182) for surgical and other therapeutic procedures. Most studies reported a similar distribution of direct medical costs. In Greece, blood transfusions constituted 38.1% of direct medical costs, and medication (45.9%) was the most important cost driver [23]. In Italy, ICT constituted 55.4% of costs, followed by transfusions (33.1%), hospitalization and surgery (3.3%), and laboratory tests and medical visits (3.1%) [26]. Another recent study confirmed that ICT was the most expensive cost component, at EUR 22,519 (70.6%), followed by blood transfusions, at EUR 6,115 (19%) [13]. In the United States, the mean annual cost of ICT was USD 19,620, which accounted for 33% of the total costs, followed by transfusions (USD 7,285), representing 12% of the total costs [29]. Another supportive study showed that direct medical costs were mainly driven by chelation therapy and blood transfusions [50]. In Iran, the annual average medication cost was USD 5026.4 (60.4%), and the annual average cost of blood transfusions was USD 1,118 (13.4%) [14]. Another study in Iran reported that the annual cost of blood transfusions per patient was EUR 581 (33.6%), and the cost of DFO was EUR 100.2 (5.8%) [24]. In India, 53% of medical costs are spent on medication [62]. In Thailand, the DFO cost was USD 199.60 (35.5%), and the blood transfusion cost was USD 106.80 (19%) [15]. In Turkey, ICT was the most expensive cost component, at USD 9,919 (69.1%), followed by in-hospital stays (USD 1,786) (12.4%) and blood transfusions (USD 1,718) (12.0%) [12]. Despite the different distributions found in previous studies, all studies concluded that ICT and blood transfusions were the main drivers of the direct medical costs of TDT.

According to ICT, the highest average annual cost per patient was estimated at AED 91,586 (USD 24,933, EUR 20,584) for combined treatment, followed by DFX at AED 88,929 (USD 24,210, EUR 19,987), DFP at AED 31,123 (USD8,473, EUR 6,995), and DFO at AED 2,058 (USD 560, EUR 463). Different estimates were found among the other studies, such as in Greece, where the mean cost per patient treated with DFX was estimated at EUR 35,928, with combined treatment at EUR 34,035, with DFO at EUR 31,637, and with DFP at EUR 17,208. This variation was due to the different combined treatments used in the two studies. In our study, combined treatments included (DFO and DFX), (DFX and DFP), (DFP and DFO), and (DFO and DFP and DFX), which accounted for the highest costs among the combined treatments. In some studies, the DFO costs were higher than our estimates because DFO was the only option available to patients at that time. In the USA, the mean annual DFO cost was USD 30,004 [25], whereas in France, the annual mean cost for DFO was EUR 16,009 [63]. The low cost of surgical and other therapeutic procedures was related to the fact that 64.3% of patients were from outside Dubai, and these patients could seek treatment for any other medical issues in the healthcare facilities near their residences. In contrast, the Dubai Thalassemia Center is a specialized center for Dubai and the Northern Emirates, which provides special treatments for TDT patients only.

In our results, direct medical costs were found to be significantly associated with age, sex, splenectomy, ICT, and the number of complications associated with TDT. These results are consistent with those of other studies that reported that increased age is associated with higher medical costs, reflecting the increased number of blood transfusions, ICT use, and inpatient hospital care in older age [10, 21, 29]. Similar to other studies [51, 54, 58], splenectomy and female sex were associated with a decrease in direct medical costs. These findings could be explained by the fact that women required less blood volume to achieve their normal hemoglobin level than male patients, thereby reducing direct medical costs. Moreover, splenectomized patients required less volume of blood transfusions than non-splenectomized patients, thereby decreasing direct medical costs. Combined therapy is associated with higher medical costs than other ICTs; in this study, this result differs from that of similar study in Greece, which estimated that DFX had the highest cost among ICTs, followed by combined therapy (DFO and DFP) [23]. A possible interpretation is that our estimates showed that combined therapy had the highest cost because it includes DFX, which has the highest price among ICTs. In our study, patients with complications had higher direct medical costs than those without complications [15, 29]. This finding could be explained by the fact that complications are associated with more HCRU, thereby increasing medical costs. Moreover, patients with multiple complications require additional healthcare resources to treat different complications simultaneously [19].

Regression analysis of direct medical costs revealed that age was a positive predictor of direct medical costs. This finding is supported by existing literature [14, 21, 29]. This could be explained by the increased number of blood transfusions with increasing age. Moreover, complications are associated with patient aging, which increases HCRU leading to increased medical costs. Deferasirox was identified as the most significant positive predictor for direct medical costs, and it caused the highest percentage change in the total direct medical costs (50%), followed by combined therapy (47%). Other studies support these findings. As reported by other studies [12, 64], DFX is associated with a significant increase in direct medical costs. Moreover, combined therapy is associated with a significant increase in direct medical costs compared to treatment with DFO [65]. These findings could be explained by the high price of DFX, which ranges from AED 785 to AED 3100 per packet according to the concentration (125–500 mg/tablet) (one packet has 28 tablets), and combined therapy that includes DFX, has a higher price than other ICTs, where the price of DFO is AED 81 per vial (2 g/vial) [66]. According to our results, the mean annual cost of combined therapy was AED 91,586, while the mean annual cost of DFO was AED 28,058.

Complications and severe serum ferritin levels (≥ 2000 ng/ml) were other positive predictors of direct medical costs. The current study revealed an expected increase in direct medical costs of 30.0% in patients with TDT complications and 11.0% in patients with severe ferritin levels (< 2000 ng/ml), compared to the absence of complications and mild-to-moderate serum ferritin levels (< 2000 ng/ml). As explained earlier, patients with complications required more blood transfusions and other medications to control their complications, and patients with severe serum ferritin levels (≥ 2000 ng/ml) had a higher number of laboratory investigations. Similar results were found in a Thai study that identified that complications and severe serum ferritin levels (≥ 2000 ng/ml) were associated with increased direct medical costs [15]. Female sex and hemoglobin E/β-thalassemia were negative predictors of direct medical costs. Female sex was associated with an expected 18% lower direct medical cost than male sex, and hemoglobin E/β-thalassemia was associated with an expected 34% lower direct medical cost. As mentioned earlier, female patients required lower volumes of blood transfusions than male patients, and there is some evidence that patients with hemoglobin E/β-thalassemia can tolerate low hemoglobin levels because of the moderate severity of hemoglobin E/β-thalassemia compared to-thalassemia, which reduces the direct medical costs [9, 51].

For direct non-medical costs, 92.5% of patients used private cars to reach their venue of treatment, Dubai Thalassemia Center. This result agrees with those of a study revealing that 92.0% of UAE residents prefer to use a private car to travel [67]. Our study revealed that the annual transportation costs were AED 2,223, which represents 1.7% of the total direct costs associated with TDT and is less than that reported in other studies. In Italy, transportation costs represent 3.4% of the total direct costs associated with TDT [26], and in Sri Lanka, transportation costs per blood transfusion visit (USD 4.26) were the second-highest cost items after food in household expenditures. Households share approximately 8% of the total cost of β-thalassemia [21]. The present study observed that 11.8% of patients experienced a catastrophic financial burden for TDT treatment, which is less than that reported in Sri Lanka and India (27.3% and 18.5%, respectively) [21, 62]. In Pakistan, a family has to spend USD 4330 to 12,987 for ten years [68], whereas in Iran, a family spends USD 25 for each blood bag and USD 280 monthly for chelation therapy [69]. In our study, most patients with catastrophic financial burdens were from a neighboring country. In Dubai, charity funding played a significant role in helping patients with low income to overcome the household cost burden associated with TDT.

## Study limitations

This study has some limitations. First, costs were estimated using a prevalence-based approach, which provides policy makers with the major cost components of TDT treatment, where the cost containment policies could be implemented. However, this type of study did not provide a complete picture of the economic costs as an incidence-based approach, which estimates the total costs throughout a patient's lifespan. Therefore, such type of studies are needed to quantify the consequences of TDT due to increasing costs of treatment with age [13, 26].

Second, the literature search identified very few published studies assessing HCRU. Hence, comparisons may not be accurate owing to different estimation tools or a lack of literature [15, 19, 21]. Further studies assessing HCRU are therefore required. These studies could help policy makers identify the most effective intervention that could reduce the frequency of HCRU, which has a significant impact on the costs of TDT treatment.

Third, there is no consensus on how to differentiate the severity of TDT thalassemia disease; thus, different criteria to evaluate thalassemia severity in different studies were found [70, 71], which may affect the quality of comparisons between studies. In this study, the patients were classified based on TDT categories, which covered β-thalassemia major, β-thalassemia intermedia, hemoglobin E/β-thalassemia, and α-thalassemia intermedia, and all these types depend on severe categories of blood transfusion. The classification of TDT thalassemia could provide detailed information on HCRU and associated direct medical costs.

Fourth, more than 60% of TDT patients lived outside Dubai and may have sought urgent medical care in their cities, leading to an underestimation of TDT costs. Connecting patients’ electronic medical records across the Emirates could solve this issue in the future.

Finally, the study was conducted in only one healthcare center; recruiting participants from more than one center and recruiting a larger sample size could improve the validity and generalizability of the results.

## Conclusions

The direct medical costs associated with TDT are high, and the management of the disease consumes various healthcare resources considerably, which are mainly predicted by age, the presence of complications, high ferritin levels, and splenectomy. Efforts must be made to improve patients' acceptance and satisfaction with their ICT to increase their compliance and improve the effectiveness of treatment, which could improve clinical outcomes and hence cost reduction. Using technology interventions would be the most beneficial choice for promoting medication adherence and disease management, such as mobile or the internet. These types of technology could encourage patients toward adherence behavior, such as reminding for the clinic visit or blood transfusion appointments or for daily consumption of medication, providing a way of communication with healthcare providers, establishing a social network for supporting patients, and providing education about disease management [72]. Hematopoietic stem cell transplantation (HSCT) is a curative therapy in patients with TDT, and it is highly cost-effective compared to conventional therapy and has long-term clinical and economic benefits that outweigh those of conventional therapy [73]. Therefore, collaboration between the Dubai Health Authority (DHA) and the Abu Dhabi Stem Cells Center (ADSCC) could positively affect TDT patients thereby improving their health outcome and the quality of life[74]. Regarding direct non-medical costs, initiating a transportation expenses program, especially for patients with financial constraints, could help with the costs of transportation associated with TDT by providing direct financial assistance, reimbursement, or vouchers.

## Supplementary Information

**Additional file 1.**

**Additional file 2.**

**Additional file 3.**

## Data Availability

The data that support the findings of this study were extracted from patients’ electronic medical records (SALAMA) in the Dubai Health Authority (DHA). Restrictions apply to the availability of these data, which were used under license for this study. Data are available from the corresponding author with permission from the Dubai Health Authority.

## Abbreviations

TDT: Transfusion-dependent thalassemia
UAE: United Arab Emirates
AED: United Arab Emirates Dirham
HCRU: Health Care Resource Utilization
ICT: Iron Chelation Therapy
DFO: Deferoxamine
DFP: Deferiprone
DFX: Deferasirox
IRR: Incidence Rate Ratio
GLM: Generalized Linear Model
