# Supplementary material for: Healthcare resource utilization and direct costs of transfusion-dependent thalassemia patients in Dubai, United Arab Emirates: a retrospective cost-of-illness study
Source: BMC Health Serv Res. 2022 Mar 5;22:304. doi: 10.1186/s12913-022-07663-6 (PMC8897869; doi:10.1186/s12913-022-07663-6)
Supplement: Supplementary file 1 — Additional file 1. [file 12913_2022_7663_MOESM1_ESM.docx]

**Transportation Costs Questionnaire- English Version**

**Direct non-medical cost (transportation costs) associated with transfusion-dependent thalassemia**

**Section 1: Demographic and socioeconomic information**

1. What is the date while you are filling in this questionnaire?

|  |  |
| --- | --- |

Day month year

|  |  |
| --- | --- |

|  |  |  |  |
| --- | --- | --- | --- |

1. What is your date of birth?

Day month year

|  |  |  |  |  |  |  |  |  |  |
| --- | --- | --- | --- | --- | --- | --- | --- | --- | --- |

1. What is your gender?

Male

Female

1. What is your nationality?

UAE

Other, please specify ----------------------------

1. What is your employment status?

Studying

Employed

Unemployed

Other, please specify -----------------------------

1. What is your monthly income/ household income in case of non-working?

------------------------AED

**Section 2: Patient transportation costs**

1. Over the last month, how many times have you visited the thalassemia center?

Number of times ----------------

1. When you visited the thalassemia center, how did you normally travel? (please circle the word that best describes how you normally traveled from your home to the thalassemia center)

- Train/metro
- Taxi
- Private car
- Other (please specify) ----------------------------------

1. If you normally traveled by public transport (bus or train/metro) for part or the entire journey, what was the cost of the two-way fares?

Cost of two-way fares (AED)-----------

1. If you normally traveled by taxi for part or the entire journey, what was the cost of the two-way fares?

Cost of two-way fares (AED)-------------

1. If you normally traveled by private car for part or the entire journey, how many kilometers did you travel two-way? If you can’t estimate the distance, please specify your city, Emirate, and the nearest landmark.

Number of kilometers (one-way) ----------------

City -------------------------

Emirate ----------------------

The nearest landmark ------------------------------

## Transportation Costs Questionnaire – Arabic Version

**التكاليف المباشره الغير طبية (تكاليف المواصلات) المرتبطة بمرض الثلاسيميا المعتمد على نقل الدم**

**أولاً: البيانات الديمغرافية**

1. ماهو تاريخ تعبئة هذا الاستبيان؟

اليوم الشهر العام

|  |  |  |  |  |  |  |  |  |  |
| --- | --- | --- | --- | --- | --- | --- | --- | --- | --- |

1. ما هو تاريخ ميلادك؟

اليوم الشهر العام

|  |  |  |  |  |  |  |  |  |  |
| --- | --- | --- | --- | --- | --- | --- | --- | --- | --- |

1. ماهو جنسك؟

ذكر

أنثى

1. ما جنسيتك؟

إماراتي

جنسية أخرى، يرجى تحديدها ----------------------------

1. ما وظيفتك؟ ضع علامة × في المربع الذي يمثل عملك المعتاد

أدرس

موظف

عاطل عن العمل

أنا أقوم بعمل آخر، اذكره -----------------------------------------

1. كم دخلك الشهري/ دخل الأسرة في حالة عدم العمل؟ ..................درهم إماراتي

**ثانياً: تكاليف المواصلات**

1. **خلال الشهر الماضي**، كم عدد مرات زيارتك لمركزعلاج الثلاسيميا؟

عدد المرات: ..............

1. عند زيارتك لمركز علاج الثلاسيميا، ماهي وسيلة النقل التي تستخدمها في المعتاد؟ (ضع علامة √ على الكلمة التي تصف وسيلة النقل التي تستخدمها في المعتاد عند انتقالك من المنزل إلى مركز علاج الثلاسيميا)

القطار/ المترو

سيارة أجرة (التاكسي)

سيارة خاصة

وسيلة أخرى (يرجى التحديد) ...........................

1. إذا كنت تنتقل عادةً بإستخدام وسيلة نقل عامة ( حافلة او قطار/مترو) في جزء من رحلتك أو لرحلتك كلها، فكم كانت تكلفة الإنتقال ذهاباً وإياباً في المرة الواحده؟

تكلفة الأجرة (ذهاباً وإياباً):................درهم إماراتي

1. إذا كنت تنتقل عادةً بإستخدام سيارة أجرة (التاكسي) في جزء من رحلتك أو لرحلتك كلها، فكم كانت تكلفة الإنتقال ذهاباً وإياباً في المرة الواحده؟

تكلفة الأجرة (ذهاباً وإياباً): ...................درهم إماراتي

1. إذا كنت تنتقل عادةً باستخدام سيارة خاصة في جزء من رحلتك أو لرحلتك كلها، كم عدد الكيلومترات التي قطعتها ذهاباً وإياباً في المره الواحدة؟ إذا لم تستطع تقدير المسافة، الرجاء تحديد موقع السكن (الإمارة والمدينة) وأقرب معلم للمنزل يمكن الإستدلال عليه.

عدد الكيلومترات (ذهاباً وإياباً): ....................كم

الإمارة: ....................

المدينة: ....................

المدينة: ....................

أقرب معلم للمنزل يمكن الإستدلال عليه: ............................

**Informed Written Consent Form: Patient agreement to participate in the study- Arabic version**

**تقدير الأعباء الإقتصادية والصحية لمرض الثلاسيميا المعتمد على نقل الدم في دولة الإمارات العربية المتحدة**

اسمي شيخة إبراهيم الشامسي، وأنا باحثة في مرحلة الدكتوراة في جامعة حمدان بن محمد الذكية، وأتناول من خلال رسالتي للدكتوراه موضوع "تقدير الأعباء الإقتصادية والصحية لمرض الثلاسيميا المعتمد على نقل الدم في دولة الإمارات العربية المتحدة"، وفي سبيل ذلك فإني أدعوكم بصفتكم **أحد المرضى البالغين والمصابين بمرض الثلاسيميا المعتمد على نقل الدم** للمشاركة في هذه الدراسة البحثية من خلال تعبئة الاستبيان المرفق.

يتكون الإستبيان من 5 أجزاء ويتطلب استكماله من 15 إلى 20 دقيقة تقريبًا، ولا يوجد أي مقابل مادي نظير الإجابة على هذا الاستبيان، كما لا يترتب على المشاركة أي مخاطر معلومة، ولضمان سرية جميع المعلومات يرجى عدم كتابة الإسم على الاستبيان.

في حالة إختياركم للمشاركة في هذا المشروع، يرجى منكم الإجابة على جميع الأسئلة بأكبر قدر ممكن من المصداقية وإعادة الاستبيان بعد الإنتهاء من تعبئته، تعتبر المشاركة في هذا الاستبيان تطوعية ويمكنكم رفض المشاركة في أي وقت.

نشكركم على وقتكم المبذول لمساعدتي في جهودي البحثية التعليمية، حيث ستوفر البيانات التي سيتم جمعها معلومات مهمة عن تأثير مرض الثلاسيميا المعتمد على نقل الدم على ميزانية الأسرة والمجتمع في دولة الإمارات العربية المتحدة من خلال تقدير التكاليف المرتبطة برعاية مريض الثلاسيميا المعتمد على نقل الدم.

تعبئة الإستبيان وإرساله إلينا دليل على موافقتكم على المشاركة في هذه الدراسة، وعند الحاجة لأي معلومات أخرى أو إن كانت لديكم أي أسئلة، يرجى التواصل معي من خلال البريد الإلكتروني الموضح أدناه.

وتفضلوا بقبول فائق الاحترام والتقدير،

الباحث الرئيسي: شيخة إبراهيم الشامسي

البريد الإلكتروني: [Shaikha_alshamsi@hbmsu.ac.ae](mailto:Shaikha_alshamsi@hbmsu.ac.ae)

المشرف الرئيسي على الرسالة: أ.د/ سامر حمايدي

البريد الإلكتروني: [s.hamidi@hbmsu.ac.ae](mailto:s.hamidi@hbmsu.ac.ae)

**Informed Written Consent Form: Patient agreement to participate in the study- English version**

**Estimating the economic and health burden of transfusion dependent thalassemia in the United Arab Emirates**

My name is Shaikha Ebrahim Alshamsi, and I am a Ph.D. student at Hamdan bin Mohamed Smart University. For my Ph.D. thesis, I am addressing the subject of “estimating the health and economic burden of transfusion dependent thalassemia in the United Arab Emirates. In this regard and in your capacity as **an adult patient with transfusion-dependent thalassemia**, I am inviting you to participate in this research study by filling out the attached survey.

The following questionnaire consists of 5 sections, and it requires approximately 15 to 20 minutes to be completed. There is neither compensation for responding nor any known risk. In order to ensure the confidentiality of all information, do not include your name in it.

If you choose to participate in this project, please answer all questions as honestly as possible and return back the completed questionnaire. Participation is strictly voluntary, and you may refuse to participate at any time.

Thank you for taking the time to assist me in my educational endeavors. The data collected will provide useful information regarding the impact of the transfusion-dependent thalassemia disease on the family budget and society in the United Arab Emirates by estimating the costs associated with caring for the patient with transfusion-dependent thalassemia.

Completion and return of the questionnaire will indicate your willingness to participate in this study. If you require additional information or have questions, please contact me at the email listed below.

Yours sincerely,

Principle investigator: Shaikha Ebrahim Alshamsi

Email: [Shaikha_alshamsi@hbmsu.ac.ae](mailto:Shaikha_alshamsi@hbmsu.ac.ae)

Main Supervisor: Prof. Samer Hamidi

Email: [s.hamidi@hbmsu.ac.ae](mailto:s.hamidi@hbmsu.ac.ae)

**Informed Written Consent Form: Parental agreement for child participate in the study- Arabic version**

**تقدير الأعباء الإقتصادية والصحية لمرض الثلاسيميا المعتمد على نقل الدم في دولة الإمارات العربية المتحدة**

اسمي شيخة إبراهيم الشامسي، وأنا باحثة في مرحلة الدكتوراة في جامعة حمدان بن محمد الذكية، وأتناول من خلال رسالتي للدكتوراه موضوع "تقدير الأعباء الإقتصادية والصحية لمرض الثلاسيميا المعتمد على نقل الدم في دولة الإمارات العربية المتحدة"، وفي سبيل ذلك فإني أدعوكم بصفتكم **أحد المرضى الأطفال أو أحد والدي طفل مصاب بمرض الثلاسيميا المعتمد على نقل الدم** للمشاركة في هذه الدراسة البحثية من خلال تعبئة الاستبيان المرفق.

يتكون الإستبيان من 4 أجزاء ويتطلب استكماله من 10 إلى 15 دقيقة تقريبًا، ولا يوجد أي مقابل مادي نظير الإجابة على هذا الاستبيان، كما لا يترتب على المشاركة أي مخاطر معلومة، ولضمان سرية جميع المعلومات يرجى عدم كتابة الإسم على الاستبيان.

في حالة إختياركم للمشاركة في هذا المشروع، يرجى منكم الإجابة على جميع الأسئلة بأكبر قدر ممكن من المصداقية وإعادة الاستبيان بعد الإنتهاء من تعبئته، تعتبر المشاركة في هذا الاستبيان تطوعية ويمكنكم رفض المشاركة في أي وقت.

نشكركم على وقتكم المبذول لمساعدتي في جهودي البحثية التعليمية، حيث ستوفر البيانات التي سيتم جمعها معلومات مهمة عن تأثير مرض الثلاسيميا المعتمد على نقل الدم على ميزانية الأسرة والمجتمع في دولة الإمارات العربية المتحدة من خلال تقدير التكاليف المرتبطة برعاية مريض الثلاسيميا المعتمد على نقل الدم.

تعبئة الإستبيان وإرساله إلينا دليل على موافقتكم على المشاركة في هذه الدراسة، وعند الحاجة لأي معلومات أخرى أو إن كانت لديكم أي أسئلة، يرجى التواصل معي من خلال البريد الإلكتروني الموضح أدناه.

وتفضلوا بقبول فائق الاحترام والتقدير،

الباحث الرئيسي: شيخة إبراهيم الشامسي

البريد الإلكتروني: [Shaikha_alshamsi@hbmsu.ac.ae](mailto:Shaikha_alshamsi@hbmsu.ac.ae)

المشرف الرئيسي على الرسالة: أ.د/ سامر حمايدي

البريد الإلكتروني: [s.hamidi@hbmsu.ac.ae](mailto:s.hamidi@hbmsu.ac.ae)

**Informed Written Consent Form: Parental agreement for child participation in the study- English version**

**Estimating the economic and health burden of transfusion dependent thalassemia in the United Arab Emirates**

My name is Shaikha Ebrahim Alshamsi, and I am a Ph.D. student at Hamdan bin Mohamed Smart University. For my Ph.D. thesis, I am addressing the subject of “estimating the health and economic burden of transfusion dependent thalassemia in the United Arab Emirates. In this regard and in your capacity as a **child patient or a parent of a child patient with transfusion-dependent thalassemia**, I am inviting you to participate in this research study by filling out the attached survey.

The following questionnaire consists of 4 sections, and it requires approximately 15 to 20 minutes to be completed. There is neither compensation for responding nor any known risk. In order to ensure the confidentiality of all information, do not include your name in it.

If you choose to participate in this project, please answer all questions as honestly as possible and return back the completed questionnaire. Participation is strictly voluntary, and you may refuse to participate at any time.

Thank you for taking the time to assist me in my educational endeavors. The data collected will provide useful information regarding the impact of the transfusion-dependent thalassemia disease on the family budget and society in the United Arab Emirates by estimating the costs associated with caring for the patient with transfusion-dependent thalassemia.

Completion and return of the questionnaire will indicate your willingness to participate in this study. if you require additional information or have questions, please contact me at the email listed below.

Yours sincerely,

Principle investigator: Shaikha Ebrahim Alshamsi

Email: [Shaikha_alshamsi@hbmsu.ac.ae](mailto:Shaikha_alshamsi@hbmsu.ac.ae)

Main Supervisor: Prof. Samer Hamidi

Email: [s.hamidi@hbmsu.ac.ae](mailto:s.hamidi@hbmsu.ac.ae)
