# Supplementary material for: Healthcare resource utilization and direct costs of transfusion-dependent thalassemia patients in Dubai, United Arab Emirates: a retrospective cost-of-illness study
Source: BMC Health Serv Res. 2022 Mar 5;22:304. doi: 10.1186/s12913-022-07663-6 (PMC8897869; doi:10.1186/s12913-022-07663-6)
Supplement: Supplementary file 2 — Additional file 2. [file 12913_2022_7663_MOESM2_ESM.docx]

**Statistical analysis and assumptions**

For comparison of continuous variables with non-normally distribution, the Mann-Whitney U test (for two groups) or Kruskal -Wallis H test (for multiple groups) was used. Chi-square test used to study the association between two categorical variables. To study the effect size of a chi-square independence test, Cramer’s V test (for nominal variables) was used. Spearman correlation was used to study the association between continuous non-normally distributed variables, and Goodman and Kruskal's lambda was used to determine the association between two nominal variables. Cramer’s V, Lambda, or Spearman correlation takes values between 0 and 1 (Table 1 and 2).

Table 1: Interpretation table of Spearman Rank-Order correlation coefficient

| Spearman correlation | Correlation |
| --- | --- |
| ≥ 0.70 | Strong relationship |
| 0.40 - 0.69 | Moderate relationship |
| 0.10 - 0.39 | Weak relationship |
| Zero | No or negligible relationship |

Source: Adapted from (1){Dancey, 2007 #1}.

Table 2: Interpretation of Cramer’s V test

| Cramer’s V | Interpretation |
| --- | --- |
| >0.25 | Very strong |
| >0.15 | Strong |
| >0.10 | Moderate |
| >0.05 | Weak |
| >0 | No or very weak |

Source: Adapted from (2).

Regression analysis was implemented to determine the factors affecting the different types of economic costs associated with TDT. Appropriate variables selection was dependent on the following strategies:

1. Variables that showed significance (p <0.25) in the bivariate analysis, {Chowdhury, 2020 #4}as well as those that were important according to the literature search (3), were included in the regression analysis.
2. The stepwise selection method was also used as a variable selection technique; it combines forward and backward procedures to add and remove variables at different steps. The cut-off value was (0.20) for removing the variable and (0.15) for adding the variable. ‘STEPWISE’ command on STATA was used (4). The appropriate tests were used to check all the following assumptions of regression analysis:
3. Sample size

Appropriate sample size affects the generalizability of the result. Fifteen subjects per independent variable are needed for a reliable equation (5). In this study, the sample size was 255, which is appropriate for the result's generalizability.

The following formula for calculating the needed variables for the study was recommended (5): N > 50 + 8m (where m = the number of required independent variables), and the sample size in this study exceeded the required number.

1. Multicollinearity and singularity

Multicollinearity could exist if two or more independent variables are highly correlated (r= 0.9 and above). In this study, multicollinearity was tested using the ‘VIF’ command; if the VIF value is more than 5, the correlation matrix was checked and removing the variables with a high correlation.

Singularity could exist when one independent variable is a combination of other independent variables, and the correlation was 1.0 or -1.0. In this study, the singularity was checked by the correlation matrix, and no cases were founded.

1. Outliers

Outliers defines as those values with standardized residual values above 3.3 or less than -3.3 and values greater than the chi-square critical values according to the number of independent variables included in the regression (Mahalanobis distance) (5). In this study, outliers were checked by the scatterplot, Mahalanobis distance test, and values greater than the chi-square critical values were excluded from the data set. Furthermore, the ‘NB_ADJUST’ command was used in negative binomial regression to check and adjust the outliers by replacing their values with random draws from a negative binomial distribution with parameters estimated using outliers' original values. Replacement values are ordered by size to preserve the outlying cases' rank order (6).

1. Normality and homoscedasticity

Normality was checked by histograms, Q-Q plots, and the Shapiro-Wilk test, which provide greater power than the Kolmogorov-Smirnov test (7). Post-estimation of normality of residuals was checked. k-density graph ‘KDENSITY R, NORMAL’ or ‘SWILK’ command in STATA used to check the normality of residuals by Shapiro-Wilk test. Logarithmic transformation was done to normalize the distribution, or other appropriate distributions were used in skewed data. Graphical and non-graphical methods were used to check homoscedasticity. For the graphical method, the residuals versus predicted values were plotted ‘RVPPLOT’ command was used in STATA, and if data points were shifted towards the right end (cone shape), it indicated heteroscedasticity. In the non-graphical methods, the white’s test (Cameron & Trivedi's decomposition of IM-test) and Breusch-Pagan/ Cool-Weisberg test for heteroskedasticity were used. Both test the null hypothesis that the variance of the residuals is homogenous. These two tests were sensitive to normality, so both methods (graphical and non-graphical) were used. According to both methods, if there was evidence of heteroscedasticity, the robust standard error was used to overcome this violation.

**Health resource utilization and direct medical costs**

The healthcare resource utilization was obtained from the electronic medical records system for each patient. According to the type of services used (laboratory investigations, radiology and other diagnostic investigations, consultations, and blood transfusion) per patient, all resources' summation was calculated.

A negative binomial regression model was conducted as the appropriate model in discrete data with over-dispersion to identify the predictors associated with healthcare resource utilization. Two ways tested overdispersion of variables: estimating the conditional mean and variances of dependent and independent variables, if variances within each group of independent were higher than the mean within each group in the independent variable, it suggested that the overdispersion was presented. The second way was by the command ‘OVERDISP’ in STATA. If the test was statistically significant, it indicated overdispersion in the data, and it was evident that the distribution of data was negative binomial distribution (8).

The negative binomial distribution is considered the most appropriate option for healthcare resource utilization (HCRU) data analysis to address the overdispersion (9). Socio-demographic and clinical characteristics were used as the main independent variables in the regression models. The following independent variables were included in the HCRU regression models according to the stepwise selection method and significant in univariate analyses or importance according to literature search:

| Dependent variables | Independent variables |
| --- | --- |
| Laboratory investigations | Disease type, patient’s age, ferritin level |
| Radiology investigations | Patients’ nationality and age |
| Blood transfusion | Complications, patients’ age, gender, splenectomy, and disease type |
| Consultations | ICT, gender, splenectomy |

The following regression models were used:

Laboratory investigations:

Log (laboratory invest.) = Intercept + b_1_(disease type) + b_2_(age) + b_3_(ferritin level)

Laboratory investigations = exp (intercept + b_1_(disease type) + b_2_(age) + b_3_(ferritin level))

Laboratory investigations= exp (Intercept) * exp (b_1_(disease type)) * exp (b_2_(age)) * exp (b_3_(ferritin level))

Radiology and other diagnostic investigations

Log (radiology investigations) = Intercept + b_1_(nationality) + b_2_(age)

Radiology investigations= exp (Intercept) * exp (b_1_(nationality)) * exp (b_2_(age))

Blood transfusion:

Log (blood transfusion) = Intercept + b_1_(complications) + b_2_(age) + b_3_(splenectomy) + b_4_(gender) + b_5_(disease type)

Blood transfusion = exp (Intercept) * exp (b_1_(complications)) * exp (b_2_(age)) * exp (b_3_(splenectomy)) * exp (b_4_(gender)) * exp (b_5_(disease type))

Consultations:

Log(consultations) = Intercept + b_1_(ICT) + b_2_(gender) + b_3_(splenectomy)

Consultations = exp (Intercept) * exp(b_1_(ICT)) * exp(b_2_(gender)) * exp (b_3_(splenectomy))

The direct medical cost was calculated by multiplying the total amount of each medical resource by the unit cost. The summation of costs from all resources utilized was considered to comprise all medical costs for a patient. The average annual cost per patient was calculated by dividing the total annual costs by the total number of patients included in the study.

A generalized linear model with log link function and gamma distribution was selected to study the significant factors affecting TDT's direct medical costs. Socio-demographic and clinical characteristics were used as the independent variables in the regression models. The following independent variables were included in the generalized linear model according to the stepwise selection method and significant in univariate analyses or importance according to literature search: age, gender, ICT, disease types, presence of complications, and ferritin level.

The following regression model was used:

Log (Direct medical costs) = Intercept + b_1_(age) + b_2_(gender) + b_3_(ICT) + b_4_(disease type) + b_5_(complications) + b_6_(ferritin level)

Direct medical costs = exp (Intercept) * exp(b_1_(age)) * exp (b_2_(gender)) * exp (b_3_(disease type) * exp (b_4_(disease type) * exp (b_5_(complications) * b_6_(ferritin level)

**Direct non-medical costs (transportation cost)**

The average number of visits, the mode of transport used, and the journey distance were obtained for all patients. For the public transport used, the fare was used to calculate the transportation cost per visit, then multiplied the cost by the total number of visits per year to calculate the annual transportation cost (equation 1).

*The annual transportation cost/ patient (public transport) = the fare of the transportation per visit x the total number of visits / year Eq. (1)*

For travel by private car, the transportation cost per visit was calculated from the journey distance in miles multiplied by an average cost per mile, allowing for fixed costs, depreciation, and running costs (equation 2). The transportation cost per visit was multiplied by the number of visits per year to find the private car's annual transportation costs (equation 3).

*The transportation cost / visit / patient (private transport) = the journey distance in miles x the average cost per mile Eq. (2)*

*The annual transportation cost / patient / year (private transport) = the transportation cost/ visit/ patient x the number of visits / year Eq. (3)*

No data were available for the UAE or neighboring countries on the average cost of driving per mile, so the data was adopted from American Automobile Association data (10); an average figure of 61.88 cents/ mile was used, which is equal to 2.27 AED/mile (based on 15,000 miles/year).

In addition, the out-of-pocket cost burden was calculated to assess any financial catastrophe on patients associated with TDT. WHO has defined the financial catastrophic as out-of-pocket payments exceeding 40% of household income net of subsistence needs (11). The World Bank uses a recent definition, which defines the financial catastrophe as out-of-pocket payments exceeding 10% of the total household income (12).

To calculate the ratio of out-of-pocket household payments for healthcare (transportation costs) to household income, the following formula is used:

*Annual household out-of-pocket expenditure for healthcare (transportation cost)/ total annual household income * 100*

Seventeen patients reported zero income and were excluded from the calculation as their income denominators were zero.

**References:**

1. Dancey CP, Reidy J. Statistics without maths for psychology: Pearson education; 2007.

2. Akoglu H. User's guide to correlation coefficients. Turkish journal of emergency medicine.2018;18(3):91-3.

3. Chowdhury MZI, Turin TC. Variable selection strategies and its importance in clinical prediction modelling. Family medicine and community health.2020;8(1).

4. Hosmer Jr DW, Lemeshow S, Sturdivant RX. Applied logistic regression: John Wiley & Sons; 2013.

5. Tabachnick BG, Fidell LS, Ullman JB. Using multivariate statistics: pearson Boston, MA; 2007.

6. Enzmann D. NB_ADJUST: Stata module to identify and adjust outliers of a variable assumed to follow a negative binomial distribution.2015.

7. Vetter TR. Fundamentals of research data and variables: the devil is in the details. Anesthesia & Analgesia.2017;125(4):1375-80.

8. Fávero LP, Belfiore P. OVERDISP: Stata module to detect overdispersion in count-data models using Stata.2018.

9. Salasar LEB, Leite JG, Neto FL. A generalized negative binomial distribution based on an extended Poisson process. Brazilian Journal of Probability and Statistics.2010;24(1):91-9.

10. American Automobile Association. YOUR DRIVING COSTS, How Much Are You Really Paying to Drive? ; 2019 2019. Available from: <https://www.aaa.com/AAA/common/AAR/files/AAA-Your-Driving-Costs.pdf>.

11. Organization WH. Designing health financing systems to reduce catastrophic health expenditure. World Health Organization; 2005.

12. World Health Organization. Monitoring the building blocks of health systems: a handbook of indicators and their measurement strategies: World Health Organization; 2010. Available from: <https://www.who.int/healthinfo/systems/WHO_MBHSS_2010_full_web.pdf>.
