# Supplementary material for: Healthcare resource utilization and direct costs of transfusion-dependent thalassemia patients in Dubai, United Arab Emirates: a retrospective cost-of-illness study
Source: BMC Health Serv Res. 2022 Mar 5;22:304. doi: 10.1186/s12913-022-07663-6 (PMC8897869; doi:10.1186/s12913-022-07663-6)
Supplement: Supplementary file 3 — Additional file 3. [file 12913_2022_7663_MOESM3_ESM.docx]

**Healthcare resource utilization associated with TDT**

Table 1 : Distribution of ICT related to characteristics of TDT patients.

| Item | DFX  (N=181) | DFP  (N=11) | DFO (N=35) | Combined therapy  (N=28) | *p*-value |
| --- | --- | --- | --- | --- | --- |
| Age group | | | | |  |
| ≤18 (N=68) | 56 | 1 | 9 | 2 | 0.023^**^ |
| >18 (N=187) | 125 | 10 | 26 | 26 |  |
| Sex  Male (N=127)  Female (N=128) | 89  92 | 8  3 | 15  20 | 15  13 | 0.367^*^ |
| Ferritin level | | | | |  |
| <2000 ng/ml (N=95) | 79 | 7 | 7 | 2 | <0.001^**^ |
| ≥2000 ng/ml (N=160) | 102 | 4 | 28 | 26 |  |
| Disease type  ꞵ-thalassemia major (N=231)  ꞵ-thalassemia intermedia (N=14)  Hemoglobin H/ꞵ-thalassemia (N=8)  α-thalassemia intermedia (N=2) | 159  14  6  2 | 10  0  1  0 | 34  0  1  0 | 28  0  0  0 | 0.412^**^ |
| Complications  No (N=44)  Yes (N=211) | 34  147 | 0  11 | 8  27 | 2  26 | 0.157^**^ |
| Splenectomy  No (N=229)  Yes (N=26) | 163  18 | 10  1 | 31  4 | 25  3 | 0.955^**^ |

* Chi-square test, ** Fisher’s exact test

Note: DFX: Deferasirox, DFP: deferiprone, DFO: deferoxamine

Table 2: Annual median healthcare resource utilization per patient related to patients characteristics (N=225).

| Item | | | Laboratory | | Radiology | | | | Blood transfusion | | | | | Consultations |
| --- | --- | --- | --- | --- | --- | --- | --- | --- | --- | --- | --- | --- | --- | --- |
| Age group | | | | | | | | | | | | | | |
| ≤18 (N=68)  >18 (N=187) | | | 122  130 | | | | 111  134 | | | 73  119 | | | 119  131 | |
| *p-value** | | | 0.416 | | | | 0.024 | | | <0.001 | | | 0.255 | |
| Sex | | | | | | | | | | | | | | |
| Male (N= 127) | | | 115 | | | | 3 | | | 45 | | | 5 | |
| Female (N= 128) | | | 109 | | | | 3 | | | 38 | | | 7 | |
| *p-value** | | | 0.384 | | | | 0.475 | | | <0.001 | | | 0.052 | |
| Nationality | | | | | | | | | | | | | | |
| UAE (N= 88) | | | 112 | | | | 2 | | | 42 | | | 5 | |
| Non-UAE (N=167) | | | 113 | | | | 3 | | | 41 | | | 6 | |
| *p-value** | | | 0.665 | | | | 0.001 | | | 0.673 | | | 0.160 | |
| Disease type | | |  | | | |  | | |  | | |  | |
| ꞵ-thalassemia major (N=231) | | | 113 | | | | 3 | | | 41 | | | 6 | |
| ꞵ-thalassemia intermedia (N=14) | | | 115 | | | | 2 | | | 37 | | | 5 | |
| Hemoglobin E/ꞵ-thalassemia (N=8) | | | 87 | | | | 4 | | | 35 | | | 5 | |
| α-thalassemia intermedia (N=2) | | | 123 | | | | 3 | | | 28 | | | 13 | |
| *p-value*** | | | 0.035 | | | | 0.647 | | | 0.059 | | | 0.379 | |
| ICT |  | | | | |  | | | | |  | | | |
| DFX (N= 181) | | 113 | | 3 | | | | 41 | | | | 5 | | |
| DFP (N= 11) | | 109 | | 1 | | | | 47 | | | | 5 | | |
| DFO (N=35) | | 116 | | 4 | | | | 39 | | | | 16 | | |
| Combined therapy (N=28) | | 115 | | 4 | | | | 43 | | | | 7 | | |
| *p-value*** | | 0.349 | | 0.391 | | | | 0.025 | | | | 0.001 | | |
| Complications | |  | |  | | | |  | | | |  | | |
| No (N= 44) | | 108 | | 1 | | | | 21 | | | | 5 | | |
| Yes (N= 211) | | 113 | | 3 | | | | 42 | | | | 6 | | |
| *p-value** | | 0.139 | | < 0.001 | | | | < 0.001 | | | | 0.120 | | |
| Ferritin level | |  | |  | | | |  | | | |  | | |
| <2000 ng/ml (N=95) | | 110 | | 3 | | | | 41 | | | | 6 | | |
| ≥2000 ng/ml (N=160) | | 114 | | 3 | | | | 40 | | | | 6 | | |
| *p-value** | | 0.040 | | 0.757 | | | | 0.350 | | | | 0.364 | | |
| Splenectomy | |  | |  | | | |  | | | |  | | |
| No (N= 229) | | 111 | | 3 | | | | 42 | | | | 5 | | |
| Yes (N= 26) | | 122 | | 5 | | | | 37 | | | | 10 | | |
| *p-value** | | 0.102 | | 0.025 | | | | 0.048 | | | | 0.021 | | |

*Mann- Whitney U test, ** Kruskal-Wallis test

Note: ICT: iron chelation therapy, DFX: Deferasirox, DFP: deferiprone, DFO: deferoxamine

**Direct medical costs associated with TDT**

Table 3: Distribution of total annual direct medical costs per patient according to TDT patients' demographic and clinical characteristics

| Demographic and clinical characteristics of TDT patients | Total direct medical costs (AED)  Median | | *p*-value |
| --- | --- | --- | --- |
| Age group  ≤18 (N=68)  >18 (N=187) | 101,271  134,933 | <0.001* | |
| Sex  Male (N=127)  Female (N=128) | 136,426  117,191 | 0.004* | |
| ICT  DFX (N=181)  DFP (N=11)  DFO (N=35)  Combined therapy (N=28) | 134,933  85,729  85,270  153,059 | <0.001** | |
| Presence of complications  No (N=44)  Yes (N=211) | 87,970  134,859 | < 0.001* | |
| Splenectomy  No (N=229)  Yes (N=26) | 143,434  127,254 | 0.045* | |

* Mann-Whitney U test, **Kruskal-Wallis test.

Note: ICT: iron chelation therapy, DFX: deferasirox, DFP: deferiprone, DFO: deferoxamine

Table 4: The relationship between the direct medical cost and number of complications and patients’ age.

| Item | Spearman’s coefficient (ρ) | *P*-value* |
| --- | --- | --- |
| Direct medical costs & number of complications | 0.202 (weak) | 0.001 |
| Direct medical costs & patients’ age | 0.269 (weak) | <0.001 |

**Non-medical cost associated with TDT**

Table 5: Mode of travel to Dubai Thalassemia Center as reported by TDT patients

| **Mode of travel** | **Number of patients** | **% of the sample** |
| --- | --- | --- |
| **Taxi** | 18 | 7.1% |
| **Private car** | 236 | 92.5% |
| **Bus** | 1 | 0.4% |

Table 6: Distribution of the mean annual transportation cost depends on patients’ characteristics.

| Item | The annual transportation cost/ patient Median | *P*-value |
| --- | --- | --- |
| No. of visits per month  One visit (N=241)  Two visits (N=14) | 1,015  2,410 | 0.026* |
| Type of transportation  Private car (N=236)  Taxi (N=18)  Bus (N=1) | 1,048  1,140  228 | 0.185** |
| Residence locations  Abu Dhabi (N=14)  Dubai (N=91)  Sharjah (N=53)  Ajman (N=28)  Umm AL Quwain (N=13)  Ras Al Khaimah (N=20)  Fujairah (N=5)  Oman (N=31) | 4,229  600  880  1,319  2,064  3,755  4,026  6,428 | <0.001** |
| Annual household income quartiles (AED)  ≤ 37,200 (N=64)  >37,200 – 96,000 (N=71)  >96,000 – 192,000 (N=60)  >192,000 (N=60) | 1,320  1015  880  1,320 | 0.385** |

*Mann-Whitney U test. **Kruskal-Wallis test
